# Amniotic Membrane-Assisted Corneal Transplantation in Ocular Perforation Due to GVHD: A Case Report

**DOI:** 10.3390/jcm15020548

**Published:** 2026-01-09

**Authors:** Nicola Cardascia, Maria Gabriella La Tegola, Francesco D’Oria, Giacomo Boscia, Francesco Boscia, Giovanni Alessio

**Affiliations:** 1 UOC Oftalmologia Universitaria, Ospedale Consorziale Policlinico di Bari, Università degli Studi di Bari, 70121 Bari, Italy; 2 UOC Oftalmologia, Ente Ecclesiastico, Ospedale Generale Regionale “F. Miulli”, 70021 Acquaviva delle Fonti, Italy

**Keywords:** ocular graft-versus-host disease, amniotic membrane transplantation, corneal perforation, penetrating keratoplasty

## Abstract

**Background/Objectives:** Ocular graft-versus-host disease (oGVHD) is a chronic, immune-mediated complication of allogeneic hematopoietic stem cell transplantation that can progress to corneal ulceration or perforation. These cases are often refractory to standard therapy and present a high risk of graft failure after keratoplasty. We report a case of oGVHD-related corneal perforation successfully managed with a novel amniotic membrane-assisted “envelope” technique during corneal transplantation. **Case Report:** A 42-year-old man with chronic oGVHD and a full-thickness corneal perforation underwent urgent repair with a lamellar patch graft completely wrapped in cryopreserved amniotic membrane, followed by penetrating keratoplasty (PKP) using an amniotic membrane envelope surrounding the donor lenticule. **Results:** The amniotic membrane provided a 360° biological barrier that isolated graft antigens from the inflammatory environment while supporting epithelial healing and stromal remodeling. Despite recurrent inflammatory episodes and multiple procedures—including cataract extraction, pars plana vitrectomy, and multilayer amniotic membrane transplantation—the graft remained clear and stable at 12-month follow-up, achieving a best-corrected visual acuity of 20/40. **Conclusions:** The amniotic membrane envelope technique may represent a valuable adjunct in managing high-risk corneal perforations secondary to oGVHD. By combining immune modulation and regenerative support, this approach can enhance tectonic stability, reduce rejection risk, and promote durable surface recovery, potentially delaying or avoiding keratoprosthesis in refractory cases.

## 1. Introduction

Graft-versus-host disease (GVHD) is a major systemic complication following allogeneic hematopoietic stem cell transplantation (allo-HSCT), affecting up to 50–70% of recipients and potentially involving the skin, liver, gastrointestinal tract, and eyes [1].

Ocular GVHD (oGVHD) develops in approximately 30–60% of patients after allo-HSCT and represents a chronic, immune-mediated inflammatory condition primarily targeting the lacrimal glands, conjunctiva, and corneal epithelium [2,3]. The resulting tear-film instability, epithelial damage, and stromal thinning can progress to corneal melting or even perforation in 1–2% of cases [4]. Such events are vision-threatening and often refractory to conventional therapies such as corticosteroids, cyclosporine, punctal occlusion, and serum eye drops [5].

Amniotic membrane transplantation (AMT) has emerged as an effective adjuvant therapy in severe ocular surface diseases owing to its anti-inflammatory, antifibrotic, and epitheliotrophic properties [6]. The membrane provides a scaffold for epithelial migration and contains growth factors that promote regeneration while modulating immune cell activity [7]. In complex GVHD-related corneal ulcers and perforations, AMT can be combined with corneal grafts to enhance graft integration and reduce immunologic rejection [8].

Despite advances in ocular surface management, corneal perforation in oGVHD remains a therapeutic challenge, with high rates of graft failure and recurrent epithelial breakdown following both lamellar and penetrating keratoplasty. Standard approaches—including patch grafts, multilayer amniotic membrane transplantation, or early PKP—often fail in the setting of persistent inflammation and immune dysregulation. In this context, there is increasing interest in surgical strategies that combine tectonic reconstruction with biological modulation of the graft–host interface. However, the use of amniotic membrane as an active component integrated within the graft architecture, rather than as a simple overlay or inlay, has been scarcely described. The present report aims to describe, in a reproducible manner, a stepwise surgical approach using an amniotic membrane “envelope” technique during corneal transplantation in a patient with severe oGVHD-related perforation.

## 2. Case Presentation

A 42-year-old Caucasian male with a 5-year history of systemic GVHD following allo-HSCT for acute myeloid leukemia presented with progressive corneal ulceration of the left eye (LE).

**Figure 1:**
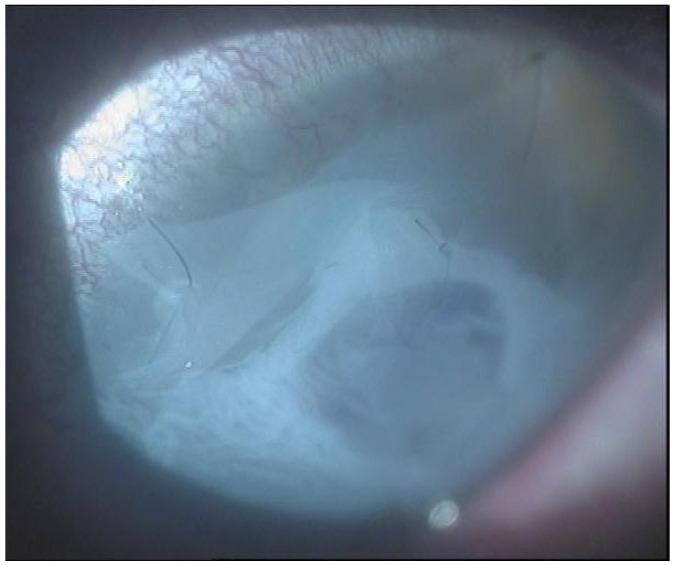
Anterior segment biomicroscopy of a round-shaped, 4 mm diameter corneal patch graft wrapped in amniotic membrane and secured with interrupted sutures.

**Figure 2:**
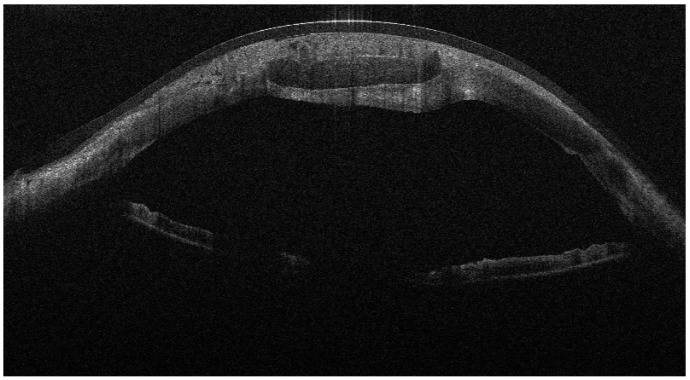
AS-OCT showing the lamellar patch graft centered on the perforation site and covered by the amniotic membrane; the AM appears as a thin hyperreflective layer over the graft surface.

From September 2023 to December 2023, the patient was managed with intensive topical antimicrobial therapy for the corneal abscess, together with aggressive ocular surface support for severe oGVHD-related epithelial instability (preservative-free lubricants, protective bandage contact lens/scleral protection when tolerated, and adjunctive therapies according to the ocular surface status). Despite these measures, progressive stromal melting occurred, culminating in a 4 mm full-thickness perforation in December 2023, prompting urgent tectonic surgery. Standardized preoperative slit-lamp photographs immediately before the urgent repair were not consistently obtainable due to the emergent nature of the procedure and the compromised ocular surface; therefore, urgent repair was performed with a lamellar corneal patch graft wrapped in cryopreserved amniotic membrane (Figure 1 and Figure 2). Cryopreserved human amniotic membrane (AM) was thawed according to the manufacturer’s instructions and rinsed with balanced saline solution. A single AM sheet was prepared to fully envelop the lamellar donor corneal patch. The donor lamellar graft was placed at the center of the AM sheet, which was then folded circumferentially to create a 360° envelope around the graft. The AM was oriented with the stromal side facing the donor corneal tissue and recipient bed, and the basement membrane/epithelial side facing outward, toward the tear film. The AM-wrapped graft was positioned over the corneal perforation and secured with interrupted 10-0 nylon sutures, ensuring complete coverage of the graft–host junction by the AM.

**Figure 3:**
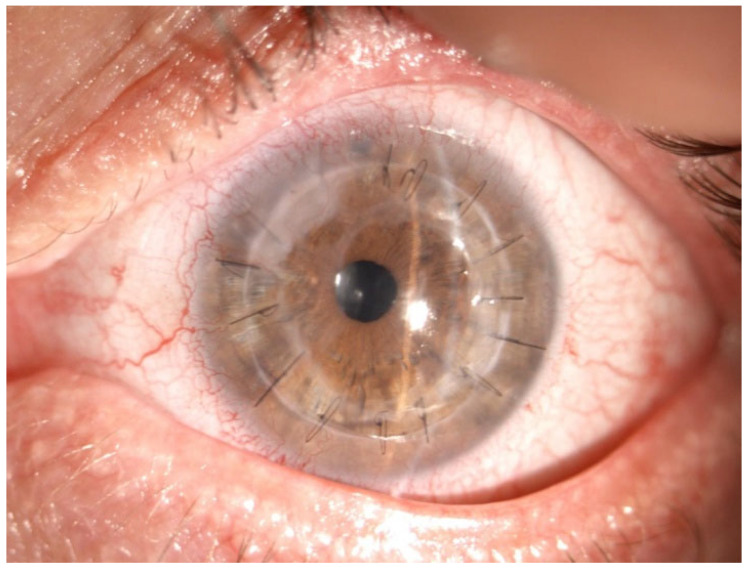
Anterior segment biomicroscopy of 8.25 mm diameter penetrating keratoplasty (PKP) with amniotic membrane overlay, 1 week after surgery.

**Figure 4:**
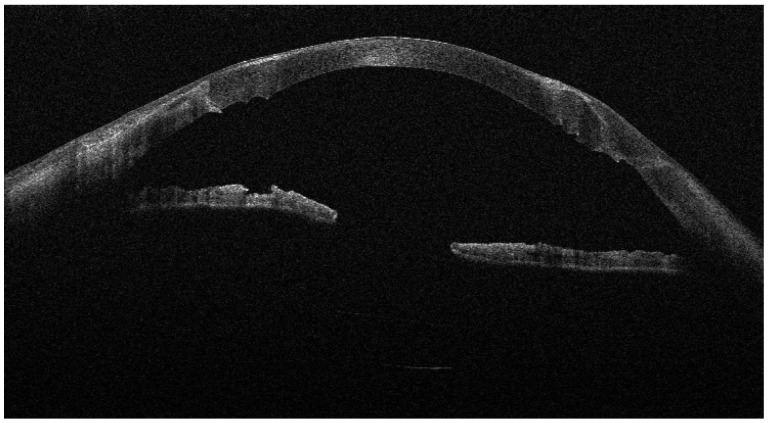
AS-OCT showing the donor tissue circumferentially surrounded by folded AM, creating a continuous ‘envelope’ at the graft–host junction.

**Figure 5:**
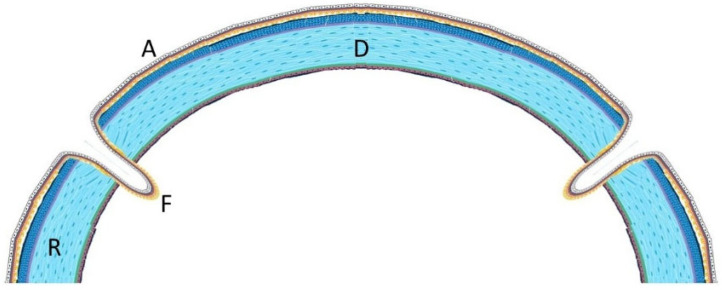
Schematic representation of the amniotic membrane (A) covering and side folding (F) to envelope the donor lenticule (D) before securing it to the recipient (R) with interrupted sutures.

Recurrent ulceration over the graft required a second patch procedure in March 2024. Subsequently, in April 2024, a full-thickness 8.25 mm PKP was performed. During penetrating keratoplasty, the donor lenticule (8.25 mm) was similarly wrapped using cryopreserved AM. The membrane was folded in a U-shaped configuration around the donor cornea, extending beyond the graft margins to cover the graft–host interface circumferentially. The same orientation was maintained (stromal side inward, epithelial side outward). The graft was then sutured to the recipient cornea with interrupted 10-0 nylon sutures, anchoring both the donor tissue and the AM envelope simultaneously. (Figure 3, Figure 4 and Figure 5).

Six months after PKP (October 2024), the patient developed a dense cataract managed by phacoemulsification with IOL implantation. Another six months later (April 2025), he presented with keratohypopyon and vitreous opacification. A 25-gauge pars plana vitrectomy (PPV) with fibrotic membrane removal and air tamponade was successfully performed.

**Figure 6:**
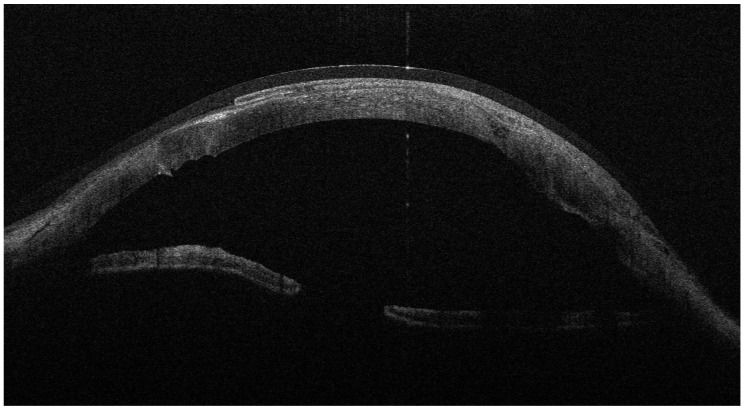
AS-OCT showing a focal micro-perforation area covered by multilayer AMT (inlay–onlay), visible as multiple hyperreflective layers sealing the defect.

In July 2025, loosened sutures caused a microabscess and micro-perforation, managed with multilayer AMT (inlay–onlay) and suture replacement (Figure 6).

**Figure 7:**
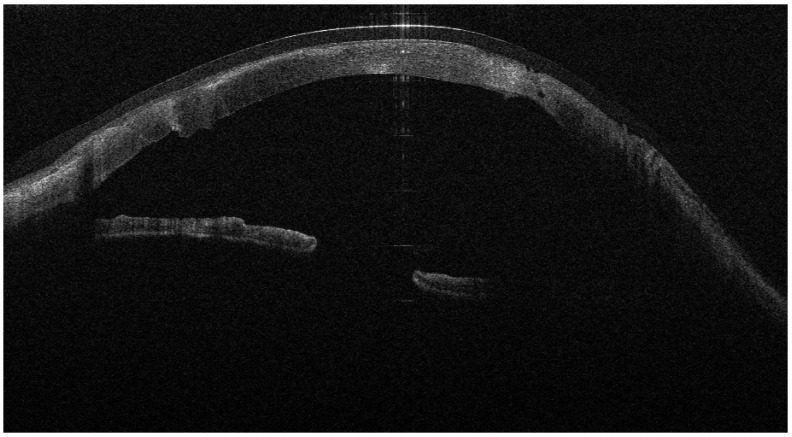
Anterior segment optical coherence tomography of spontaneous resolution of a peripheral ulcer of the donor lenticule.

A transient peripheral ulcer developed the following month but healed spontaneously (Figure 7).

**Figure 8:**
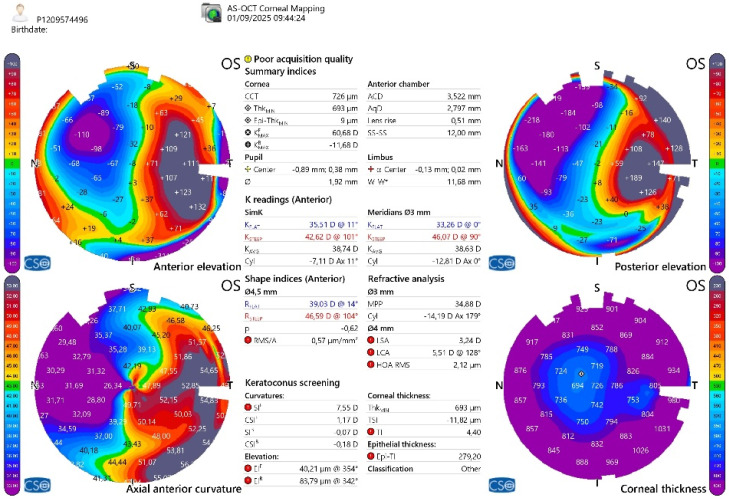
Corneal topography and pachymetry of the left eye showing residual irregular astigmatism induced by PKP.

Final follow-up (October 2025) showed a stable, clear graft with BCVA 20/40 OS and no recurrent epithelial defects (Figure 8).

The patient received topical betamethasone + chloramphenicol (QID) and a soft therapeutic bandage contact lens (16.5 mm, Regenera G-72 HW, 72% H_2_O, Aloe vera; Eye Pharma SpA, Genoa, Italy) throughout recovery, which likely contributed to ocular surface protection and epithelial stability and should be considered an adjunctive factor of the surgical technique itself.

## 3. Discussion

Ocular GVHD is among the most challenging ocular surface diseases after allo-HSCT because the persistent immune dysregulation undermines epithelial stability and stromal homeostasis, occasionally culminating in corneal perforation [1,2,3,4]. Severe and recurrent oGVHD is often a manifestation of active systemic disease. Systemic immunosuppressive therapy was managed in coordination with the hematology team according to the patient’s overall clinical status. Local surgical interventions should be considered complementary to, and not a replacement for, appropriate systemic GVHD control. In such eyes, the therapeutic goal extends beyond closing a tectonic defect: surgeons must also reconstruct a biologically favorable niche that tempers inflammation, supports epithelial migration, and protects the graft from immune exposure [1,3,5]. The anterior lamellar patch graft combined with the amniotic membrane envelope should be regarded as a tectonic, temporizing measure in high-risk oGVHD eyes rather than a definitive solution. In this context, the technique served to stabilize the cornea and maintain globe integrity before proceeding to PKP under more favorable ocular surface conditions.

This rationale guided our choice of an amniotic membrane (AM) “envelope” around the donor corneal tissue. While AM is well established as an anti-inflammatory, antifibrotic, and epitheliotrophic substrate in ocular surface disease, its value is amplified when it encases (rather than merely overlays) the donor lenticule [6,7,8]. In this configuration, AM likely functioned as a 360° biological barrier, physically separating graft antigens from the pro-inflammatory tear film and host immune effectors typical of oGVHD, thereby mitigating rejection risk [6,8,9]. In parallel, AM’s reservoir of growth and anti-scarring factors—such as EGF, KGF, and HGF—may have promoted epithelial healing and stromal remodeling, facilitating long-term optical clarity [7].

The timeline of this case also underscores the importance of surface protection (Table 1).

A therapeutic bandage/scleral lens regimen reduced shear stress, prolonged AM residence, and stabilized the ocular surface in the fragile early postoperative period—an approach supported by contemporary oGVHD management paradigms [5,10]. Notably, despite recurrent inflammatory episodes and the systemic immune background, our patient achieved a stable, clear graft with BCVA of 20/40 at long-term follow-up, suggesting that an AM-enveloped graft can be an effective bridge strategy before considering keratoprosthesis in high-risk, immune-mediated corneal disease [8,9].

More broadly, this experience supports reframing GVHD-related perforation not only as a structural failure but also as a failure of tissue homeostasis. Integrating regenerative substrates like AM within keratoplasty may harmonize host–donor interactions, dampen immune activation, and extend graft survival in oGVHD and possibly other autoimmune keratopathies [1,6,7,8,9]. Prospective studies comparing envelope-assisted techniques with standard AM overlay/inlay or conventional PKP are warranted to quantify immunologic benefits, epithelialization kinetics, and survival curves in this population [4,6,7,8,9]. Given the single-case nature of this report, no conclusions regarding safety or efficacy can be drawn. Theoretical risks of incorporating amniotic membrane within the wound interface include potential wound leakage, altered suture tension, and increased susceptibility to infection. These aspects warrant systematic evaluation in larger series.

## 4. Conclusions

In a corneal perforation driven by chronic ocular GVHD, combining penetrating keratoplasty with an amniotic membrane “envelope” achieved sustained tectonic stability and useful vision, despite a hostile inflammatory milieu. By simultaneously modulating immunity and supporting regeneration, this tailored approach may reduce rejection risk and improve surface rehabilitation compared with conventional methods [5,6,7,8,9]. Incorporation of protective contact lens strategies complements the biologic reconstruction and may further enhance outcomes in oGVHD [10]. Future research should focus on comparative studies evaluating amniotic membrane envelope-assisted keratoplasty versus conventional AM overlay/inlay techniques in high-risk corneal perforations. Key endpoints should include epithelialization time, graft survival, immune rejection rates, and the need for keratoprosthesis. Beyond oGVHD, this envelope-based approach may be explored in other immune-mediated or inflammatory corneal conditions characterized by severe surface instability, such as Stevens–Johnson syndrome or autoimmune keratolysis.

## Figures and Tables

**Table 1:** Timeline of clinical events. Abbreviations: AM, amniotic membrane; BCVA, best-corrected visual acuity; PKP, penetrating keratoplasty; PPV, pars plana vitrectomy.

Date	Intervention	Outcome
September–December 2023	Corneal ulcer → perforation	AM-wrapped patch graft
March 2024	Repeat patch graft	Temporary closed
April 2024	PKP with AM envelope	Clear graft
October 2024	Cataract surgery	Improved vision
April 2025	PPV for keratohypopion	Controlled inflammation
July 2025	AMT for microperforation	Surface re-epithelialized
October 2025	Final follow-up	BCVA 20/40

## Data Availability

The original contributions presented in this study are included in the article. Further inquiries can be directed to the corresponding author.
